# Supplementary material for: Open Pyeloplasty in Infants under 1 Year—Proven or Meaningless?
Source: Children (Basel). 2023 Jan 31;10(2):257. doi: 10.3390/children10020257 (PMC9955854; doi:10.3390/children10020257)
Supplement: Supplementary file 1 [file children-10-00257-s001.zip › children-2167404-supplementary.pdf]

Figure S1) questionnaire for parents and patients (original in german)

1. Do you agree with the survey?
   - O yes
   - O no

2. Who answers the questionaire?
   - O mother
   - O father
   - O with child
   - O without child
   - O Child allone

3. How old was the child at the time of surgery (in month)?

4. How many month ago did the surgery take place (up to 24 months in months, from 24 months in years) ?

5. How was the wound closed?
   - O Absorbable suture material
   - O Non-absorbable suture material
   - O Skin staples

6. How did the wound heal after surgery?
   - O No complications
   - O Slight redness, no pain, wound remained closed
   - O Significant redness and infection, pain, wound remained closed
   - O Wound was inflamed and secreted, wound has opend superficially, no re-operation required
   - O Wound was inflames and secreted, wound has opend, further surgery for wound closure was necessary

7. Were there any further complications after the surgery?
   - O none
   - O strong pain
   - O bleeding
   - O infection: urinary tract infection or wound infection (please underline appropriate)
   - O hernia formation (formation of a gap/protrusion in the area of the scar)
   - O other: ……….

8. Is your child currently still complaining of pain in the area of the scar?
   - O yes
   - O no

9. How does the scar currently look like?
   - ○ Fine white line, barely visible
   - ○ White line, visible, flat and level
   - ○ White line, visible, raised
   - ○ Red, raised
   - ○ bulging

10. How satisfied are you (parents) with the appearance of the scar?
   - ○ Very satisfied
   - ○ satisfied
   - ○ is okay
   - ○ dissatisfied
   - ○ very dissatified

11. How satisfied are you (patient) with the appearance of the scar? (please let your child answer)
   - ○ Very satisfied (the scar does not bother me at all)
   - ○ Satisfied (the scar hardly bothers me)
   - ○ is okay (well, the scar bothers me occasionally)
   - ○ dissatisfied (the scar bothers me)
   - ○ dissatisfied (the scar bothers me and I try to hide it)

12. Were you satisfied with the surgery overall (preparation, surgery, inpatient care)?
   - ○ Very satisfied
   - ○ satisfied
   - ○ was okay
   - ○ dissatisfied
   - ○ very dissatisfied

13. Would you like to add your own words to question 12?

14. Would you have the operation performed again as an open pyeloplasty?
   - ○ Yes
   - ○ no

Figure S2) scar classification by modified Vancouver scar scale (original in german)

vascularization:	○	normal	0
	○	pink	1
	○	red	2
	○	purple	3
pigmentation:	○	normal	0
	○	hypopigmented	1
	○	hyperpigmented	2
level:	○	flat / level	0
	○	slightly raised (< 2mm)	1
	○	fairly raised (2-5mm)	2
	○	bulging (> 5mm)	3
	○	sunken	4
		total:	________
